# 604. Impact of COVID-19 Pandemic on Telehealth Practices in Pediatric Infectious Diseases

**DOI:** 10.1093/ofid/ofab466.802

**Published:** 2021-12-04

**Authors:** Sindhu Mohandas, Daniel Olson, Sergio Fanella, Amin Hakim, Claudia Gaviria-Agudelo, Sabah Kalyoussef

**Affiliations:** 1 Children Hospital Los Angeles, Los Angeles, CA; 2 University of Colorado, Denver, CO; 3 University of Manitoba, Winnipeg, MB, Canada; 4 Emz Solutions, New York, NY; 5 University of South Florida Morsani College of Medicine, Tampa, FL; 6 The Children's Hospital at Saint Peter's University Hospital, Clinical Assistant Professor at Rutgers Robert Wood Johnson Medical School, New Brunswick, NJ

## Abstract

**Background:**

The COVID-19 pandemic has led to changes in clinical practice, including a significant increase in the use of telehealth (TH). We sought to assess the impact of the pandemic on the use and perceptions of TH by pediatric infectious diseases (PID) clinicians.

Figure 1. Modalities

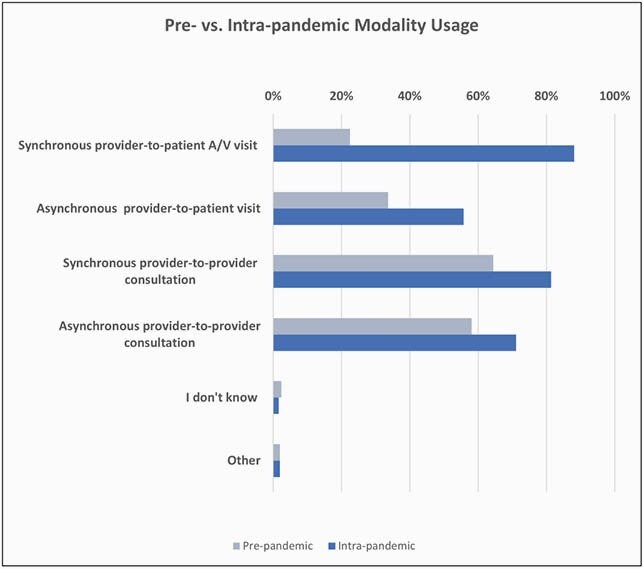

Figure 2. Comfort

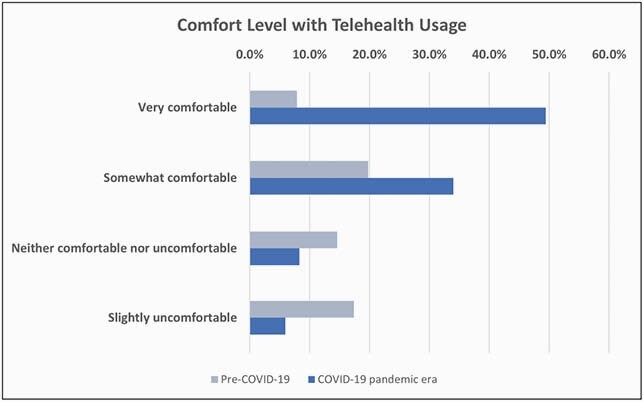

**Methods:**

The PIDS* Telehealth Working Group developed a 26-question online survey to assess telehealth practices among PID clinicians. The survey was available via Survey Monkey® from 12/6/2020-2/26/2021 to members of PIDS, PICNIC*, AAMI and AAP*. Clinicians in active practice in North America were included in the analysis.

Figure 3. Platforms

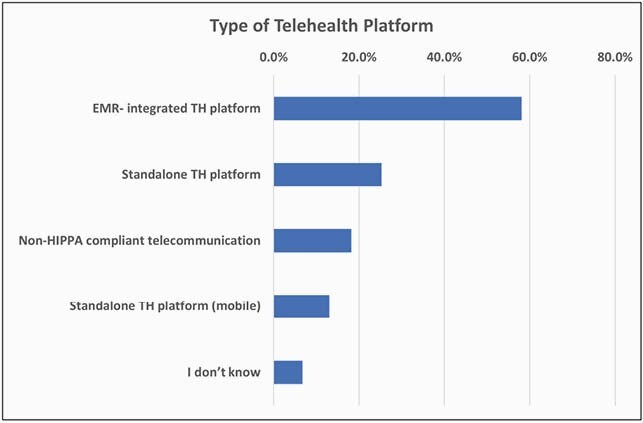

Figure 4. Barriers

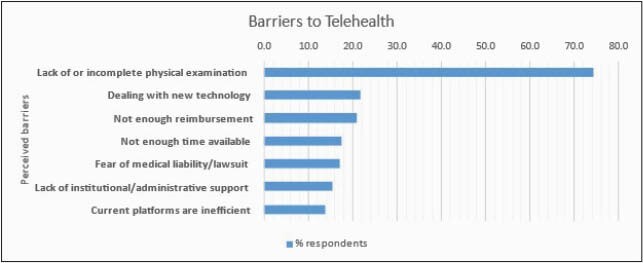

**Results:**

The response rate was 10% (n=253) of 2,550 PID clinicians. Physicians accounted for 98.4% of the cohort. The remaining 1.6% were allied health professionals. 81 survey respondents (32%) were in 4 US states (CA, TX, OH and NY) and the province of Quebec. 62.8% of respondents were women, 37% of respondents were 36-45 years old, with 42.7% devoting about 50-99% of their time to direct patient care. TH usage increased during the pandemic with the most gain in provider-patient communications with 65.6% increase for synchronous and 22.1% for asynchronous TH (Figure 1). Gains in provider-provider TH were less than 20%. Respondents reported a 6-fold gain in comfort with TH usage versus pre-pandemic level (Figure 2). Most respondents report being satisfied with their current platform and modality. Once the COVID-19 waivers expire, 70% of respondents plan to continue using TH. The most common TH modality used was an EMR-integrated TH platform (Figure 3). The main perceived barriers to TH adoption were lack of complete physical examination (73.7%), dealing with new technology (21.5%), and insufficient reimbursement (20.8%) (Figure 4).

**Conclusion:**

The COVID-19 pandemic has resulted in a significant increase in the use of TH by PID specialists versus pre-pandemic usage. Respondents gained comfort with use of different telehealth modalities during the pandemic. This data can help clinicians and organizations in planning and resource allocation for telehealth programs in a post-pandemic environment.

**Disclosures:**

**All Authors**: No reported disclosures

